# A multi-center, open-label, randomized study to explore efficacy and safety of baricitinib in active primary Sjogren’s syndrome patients

**DOI:** 10.1186/s13063-023-07087-5

**Published:** 2023-02-15

**Authors:** Wei Bai, Fan Yang, Huji Xu, Wei Wei, Hongbin Li, Liyun Zhang, Yi Zhao, Xiaofei Shi, Yan Zhang, Xiaofeng Zeng, Xiaomei Leng

**Affiliations:** 1 Department of Rheumatology and Clinical Immunology, Peking Union Medical College Hospital, Chinese Academy of Medical Sciences, Peking Union Medical College; National Clinical Research Center for Dermatologic and Immunologic Diseases (NCRC-DID), Ministry of Science & Technology; State Key Laboratory of Complex Severe and Rare Diseases, Peking Union Medical College Hospital; Key Laboratory of Rheumatology and Clinical Immunology, Ministry of Education, Beijing, 100730 China; 2 Department of Rheumatology and Immunology, Changzheng Hospital, Naval Medical University, Shanghai, 200003 China; 3 Department of Rheumatology and Immunology, Tianjin Medical University General Hospital, Tianjin, China; 4 Department of Rheumatology, The Affiliated Hospital of Inner Mongolia Medical University, Hohhot, Inner Mongolia China; 5 Department of Rheumatology, Third Hospital of Shanxi Medical University, Bethune Hospital Shanxi Academy of Medical Sciences, Taiyuan, Shanxi China; 6 Department of Rheumatology and Allergy, Xuanwu Hospital, Capital Medical University, Beijing, China; 7 Department of Rheumatology and Immunology, The First Affiliated Hospital and College of Clinical Medicine, Henan University of Science and Technology, Luoyang, Henan China; 8 Department of Rheumatology and Immunology, Tangdu Hospital, Fourth Military Medical University (Air Force Medical University), Xi’an, Shaanxi, China

**Keywords:** Primary Sjogren’s syndrome, JAK/STAT, Baricitinib, Hydroxychloroquine, ESSDAI

## Abstract

**Background:**

Primary Sjogren’s syndrome (pSS) is a systemic autoimmune disease involving multiple organ systems. The Janus kinase/signal transduction and activator of transcription (JAK/STAT) signaling pathway is a key pathway involving the pathogenesis of pSS. Baricitinib, a selective JAK1 and JAK2 inhibitor, has been approved for treatment of active rheumatoid arthritis and reported in treatment of some other autoimmune diseases including systemic lupus erythematosus. We have found that baricitinib might be effective and safe in pSS in a pilot study. However, there is no published clinical evidence of baricitinib in pSS. Hence, we conducted this randomized study to further explore the efficacy and safety of baricitinib in pSS.

**Methods:**

This is a multi-center, prospective, open-label, randomized study to compare the efficacy of baricitinib + hydroxychloroquine (HCQ) with HCQ alone in pSS patients. We plan to involve 87 active pSS patients with European League Against Rheumatism pSS disease activity index (ESSDAI) ≥ 5 from eight different tertiary centers in China. Patients will be randomized (2:1) to receive baricitinib 4 mg per day + HCQ 400 mg per day or HCQ 400 mg per day alone. We will switch HCQ to baricitinib + HCQ if the patient in the latter group has no ESSDAI response at week 12. The final evaluation will be at week 24. The primary endpoint is the percentage of ESSDAI response, or minimal clinically important improvement (MCII), which was defined as an improvement of ESSDAI at least three points at week 12. The secondary endpoints include EULAR pSS patient-reported index (ESSPRI) response, change of Physician’s Global Assessment (PGA) score, serological activity parameters, salivary gland function test, and focus score on labial salivary gland biopsy.

**Discussion:**

This is the first randomized controlled study to evaluate the clinical efficacy and safety of baricitinib in pSS. We hope that the result of this study can provide more reliable evidence of the efficacy and safety of baricitinib in pSS.

**Trial registration:**

ClinicalTrials.gov NCT05016297. Registered on 19 Aug 2021.

## Administrative information

Note: the numbers in curly brackets in this protocol refer to SPIRIT checklist item numbers. The order of the items has been modified to group similar items (see http://www.equator-network.org/reporting-guidelines/spirit-2013-statement-defining-standard-protocol-itemsfor-clinical-trials/).

**Table:** 

Title {1}	A multi-center, prospective, open-label, randomized study to explore efficacy and safety of baricitinib in active primary Sjogren’s syndrome patients
Trial registration {2a and 2b}	ClinicalTrials.gov, ID: NCT05016297
Protocol version {3}	Protocol Version 3.1, 30 January 2022
Funding {4}	This study is funded by Eli Lilly Trading Co., Ltd.
Author details {5a}	Wei Bai^1^, Fan Yang^1^, Huji Xu^2^, Wei Wei^3^, Hongbin Li^4^, Liyun Zhang^5^, Yi Zhao^6^, Xiaofei Shi^7^, Yan Zhang^8^, Xiaofeng Zeng^1^, Xiaomei Leng^1^ 1 Department of Rheumatology and Clinical Immunology, Chinese Academy of Medical Sciences & Peking Union Medical College; National Clinical Research Center for Dermatologic and Immunologic Diseases (NCRC-DID), Ministry of Science & Technology; State Key Laboratory of Complex Severe and Rare Diseases, Peking Union Medical College Hospital (PUMCH); Key Laboratory of Rheumatology and Clinical Immunology, Ministry of Education, Beijing 100,730, China 2 Department of Rheumatology and Immunology, Changzheng Hospital, Naval Medical University, Shanghai, 200,003, China. 3 Department of Rheumatology, Tianjin Medical University General Hospital, Tianjin, China. 4 Department of Rheumatology, The Affiliated Hospital of Inner Mongolia Medical University, Hohhot, Inner Mongolia, China. 5 Department of Rheumatology, Third Hospital of Shanxi Medical University, Shanxi Bethune Hospital Shanxi Academy of Medical Sciences, Taiyuan, Shanxi, China. 6 Department of Rheumatology, Xuanwu Hospital, Capital Medical University, Beijing, China. 7 Department of Rheumatology, the First Affiliated Hospital and College of Clinical Medicine, Henan University of Science and Technology, Luoyang, Henan, China. 8 Department of Rheumatology, Tangdu Hospital, Fourth Military Medical University (Air Force Medical University), Xi’an, Shaanxi, China.
Name and contact information for the trial sponsor {5b}	Dr. Xiaomei Leng Department of Rheumatology and Clinical Immunology, Chinese Academy of Medical Sciences & Peking Union Medical College; National Clinical Research Center for Dermatologic and Immunologic Diseases (NCRC-DID), Ministry of Science & Technology; State Key Laboratory of Complex Severe and Rare Diseases, Peking Union Medical College Hospital (PUMCH); Key Laboratory of Rheumatology and Clinical Immunology, Ministry of Education, Beijing 100,730, China. Tel: + 86–10-69,155,646; Email: lpumch@126.com
Role of sponsor {5c}	This is an investigator initiated clinical trial. Therefore, the funders played no role in the design of the study and collection, analysis, and interpretation of data and in writing the manuscript.

## Introduction

### Background and rationale {6a}

Primary Sjogren’s syndrome (pSS) is a systemic autoimmune disease characterized by dysfunction of the exocrine glands. The clinical hallmarks of pSS are keratoconjunctivitis sicca and xerostomia, or the sicca complex. Important organs including renal, pulmonary, or central nervous system involvement can be seen in patients with pSS. The manifestations of pSS result from a predominantly lymphocytic cell infiltration of glandular and non-glandular organs. The abnormal activation of T helper (Th) 1/Th17 lymphocytes, B lymphocytes, and plasma cells are related to the pathogenesis of pSS.

Th17 cells, as an important subset of CD4 + T cells, have been recognized in recent years. Under the stimulation of interleukin(IL)-6, transforming growth factor(TGF)-β, IL-21, IL-23, and other cytokines, CD4 + T cells can differentiate into Th17 cells, which with highly expression of RoRγt, and secrete IL-17, IL-22 and other cytokines to promote the activation of B-cells and promote the inflammatory response of target organs [1, 2]. Many literatures have found that Th17 cells and its related cytokines such as IL-17 are activated and increased in peripheral blood [3], salivary gland tissue [4], and tear [5] of patients with pSS. In a variety of Sjogren’s syndrome animal models, including C57BL/6.NOD-Aec1Aec2 mice, abnormal activation of Th17 cells, increased IL-17 levels, and infiltration of Th17 cells in salivary gland tissue were found [6]. These studies verified that Th17 cells play an important role in the pathogenesis of pSS.

Janus kinase (JAK) / (signal transducer and activator of transcription) STAT signaling pathway is activated and plays as a key pathway in the differentiation and activation of many lymphocytes, which affect the pathogenesis of many autoimmune diseases [7]. Many studies had identified that JAK/STAT signaling pathway was the key pathway of Th17 cell differentiation and activation. Genome-wide association studies (GWASs) had shown that STAT3 plays a key role in transcriptional regulation during early differentiation of Th17 cells [8]. Several in vitro and animal experiments have revealed that inhibition of JAK/STAT pathway, especially JAK1-3/STAT3 pathway, can inhibit the differentiation and function of Th17 cells in animal models including ankylosing spondylitis [9], psoriasis [10], and autoimmune arthritis [11, 12]. JAK/STAT pathway was also playing a key role in the activation of interferon (IFN), especially type I IFN, which was important in the pathogenesis of many systemic autoimmune diseases [13], such as systemic lupus erythematosus (SLE) and pSS [14]. So JAK/STAT pathway may take part in the process of many autoimmune diseases including pSS by affecting many cytokine signals.

JAK inhibitors have also been widely used in the treatment of rheumatoid arthritis (RA) and other autoimmune diseases [7]. Some studies have verified that JAK/STAT pathway is activated in patients with pSS, and JAK inhibitor may be effective for pSS. Lee et al. found that filgotinib, the selective JAK1 inhibitor, suppressed the IFN-induced transcription of differentially expressed genes and B-cell activating factor (BAFF) in human primary salivary gland epithelial cells [15]. In addition, filgotinib-treated mice exhibited increased salivary flow rates and marked reductions in the lymphocytic infiltration of salivary glands. JAK inhibitors AG490 and ruxolitinib can reverse DNA methylation and hydroxymethylation of salivary gland epidermal cells in pSS [16]. All these basic studies had shown that JAK inhibitors might be a novel therapeutic approach for pSS. A randomized phase 2 study is currently in progress to assess the safety and efficacy of filgotinib in adult subjects with active Sjogren’s syndrome (ClinicalTrials.gov ID: NCT03100942). A phase 1/2 study of JAK inhibitor tofacitinib demonstrated a trend for improving both signs and symptoms of patients with dry eye disease. So far, only one basic study has focused on baricitinib for pSS [17], in which Aota et al. demonstrated baricitinib suppressed IFN-γ induced CXCL10 expression in human salivary gland ductal cells and suggested its potential for the treatment of pSS. There is no evidence of the safety and efficacy of baricitinib in patients with pSS. We plan to conduct this study to explore the efficacy and safety of baricitinib in pSS.

### Objectives {7}

We hope to provide reliable evidence of baricitinib as a new potential oral therapy for pSS. The primary endpoint is the percentage of European League Against Rheumatism (EULAR) pSS disease activity index (ESSDAI) response, or minimal clinically important improvement (MCII), which was defined as an improvement of ESSDAI at least three points [18], at 12 weeks.

### Trial design {8}

This is a multi-center, prospective, open-label, randomized study to compare the efficacy of baricitinib + hydroxychloroquine (HCQ) with HCQ alone in pSS patients. Patients will be randomized (2:1) to receive baricitinib 4 mg per day + HCQ 400 mg per day or HCQ 400 mg per day alone. We will switch HCQ to baricitinib + HCQ if the patient in the latter group has no response at week 12. The final evaluation will be at week 24.

## Methods: participants, interventions, and outcomes

### Study setting {9}

This study will be performed in eight tertiary referral centers (Peking Union Medical College Hospital, et al.) in China. Patients are recruited at the outpatient clinic of department of rheumatology in these hospitals. Patients are considered for inclusion if they meet the criteria as defined below.

### Eligibility criteria {10}

#### Inclusion criteria

1. Must read and understand the informed consent approved by the institutional review board (IRB)/ethics review board (ERB) governing the site and provide written informed consent.
2. Stated willingness to comply with all study procedures and availability for the duration of the study.
3. Ability to take oral medication and be willing to adhere to the study intervention regimen.
4. Male or female, aged between 18 and 75 years.
5. Fulfill the 2016 ACR/EULAR classification criteria for pSS [19].
6. With moderate activity (ESSDAI ≥ 5) on HCQ 400 mg per day treatment for at least 12 weeks at the screening visit.
7. With serological activity defined as hypocompleminemia or elevated C-reactive protein (CRP)/erythrocyte sedimentation rate (ESR)/immunoglobulin G (IgG) /rheumatoid factor (RF) level (excluding acute and chronic infection and other factors).
8. Nonpregnant, nonbreastfeeding female patient.
9. Males with potential for reproduction must agree to practice effective birth control methods described above too.

#### Exclusion criteria

1. Have received any of the following medications:
  a Biologic treatments for immunologic disease such as etanercept, infliximab, certolizumab, adalimumab, golimumab, tocilizumab, abatacept, ustekinumab, ixekizumab, secukinumab, or anakinra within 4 weeks of screening. Cyclophosphamide (or any other cytotoxic agent), belimumab, or anifrolumab (or another anti-IFN therapy) within 12 weeks of screening.
  b Cyclophosphamide (or any other cytotoxic agent), belimumab, or anifrolumab (or another anti-IFN therapy) within 12 weeks of screening.
  c Rituximab, any other B-cell depleting therapies, or intravenous immunoglobulin (IVIg), or pulse methylprednisolone within 24 weeks of screening.
  d Glucocorticoids which dosage greater than 10 mg prednisone per day.
  e Methotrexate, azathioprine, mycophenolate mofetil, cyclosporine, tacrolimus within 4 weeks at the time of screening.
  f Plasmapheresis within 12 weeks of screening.
2. History of chronic liver disease or elevated liver function tests:
  a Alanine aminotransferase (ALT) or aspartate aminotransferase (AST) > 2 × upper limit of normal at screening.
  b Serum total bilirubin ≥ 1.5 × upper limit of normal at screening.
3. With estimated glomerular filtration rate (eGFR) < 40 mL/min/1.73 m2 (Bedside Schwartz formula 2009) or have received hemodialysis, peritoneal dialysis, or intestinal dialysis.
4. Protein to creatinine ratio of more than 1 mg/dL repeated and confirmed three times or confirmed with 24 h urine protein of more than 1000 mg.
5. White blood cell (WBC) < 2 × 109 cells/L or absolute neutrophil count (ANC) < 1 × 109 cells/L, hemoglobin (Hgb) < 9.0 g/dL or platelets < 1 × 109 cells/L or absolute lymphocyte count (ALC) < 0.5 × 109 cells/L.
6. Have screening laboratory test values, including thyroid-stimulating hormone (TSH), outside the reference range for the population that, in the opinion of the investigator, pose an unacceptable risk for the patient’s participation in the study. Patients who are receiving thyroxine as replacement therapy may participate in the study, provided stable therapy has been administered for ≥ 12 weeks and TSH is within the laboratory’s reference range. Patients who have TSH marginally outside the laboratory’s normal reference range and are receiving stable thyroxine replacement therapy may participate if the treating physician has documented that the thyroxine replacement therapy is adequate for the patient.
7. Pregnant or lactating women. Women of childbearing potential are required to have a negative pregnancy test at screening.
8. Have had any major surgery within 8 weeks prior to screening or will require major surgery during the study that, in the opinion of the investigator, would pose an unacceptable risk to the patient.
9. Have experienced any of the following within 12 weeks of screening: venous thromboembolism (VTE, deep vein thrombosis [DVT] /pulmonary embolism [PE]), myocardial infarction, unstable ischemic heart disease, stroke, New York Heart Association Stage III/IV heart failure, or have a history of recurrent (≥ 2) VTE (DVT/PE).
10. Have a history or presence of cardiovascular, respiratory, hepatic, gastrointestinal, endocrine, hematological, neurological, or neuropsychiatric disorders or any other serious and/or unstable illness that in the opinion of the investigator, could constitute an unacceptable risk when taking investigational product or interfere with the interpretation of data.
11. Have a history of lymphoproliferative disease; have signs or symptoms suggestive of possible lymphoproliferative disease, including lymphadenopathy or splenomegaly; have active primary or recurrent malignant disease; or have been in remission from clinically significant malignancy for < 5 years prior to randomization. 
  The following may be exempted:
  a Patients with cervical carcinoma in situ that has been resected with no evidence of recurrence or metastatic disease for at least 3 years may participate in the study.
  b Patients with basal cell or squamous epithelial skin cancers that have been completely resected with no evidence of recurrence for at least 3 years may participate in the study.
12. Have a current or recent (< 4 weeks prior to randomization) clinically serious viral, bacterial, fungal, or parasitic infection or any other active or recent infection that in the opinion of the investigator, would pose an unacceptable risk to the patient if participating in the study.
13. Have symptomatic herpes simplex at the time of randomization, a history of disseminated/complicated herpes zoster, or have had symptomatic herpes zoster infection within 12 weeks prior to randomization.
14. Have a positive test for hepatitis B virus (HBV) defined as:
  a Positive for hepatitis B surface antigen (HBsAg), or
  b Positive for hepatitis B core antibody (HBcAb) and positive for hepatitis B virus deoxyribonucleic acid (HBV DNA).
     Note: Patients who are HBcAb-positive and HBV DNA-negative may be enrolled in the study but will require additional HBV DNA monitoring during the study.
15. Have hepatitis C virus (HCV) infection (hepatitis C antibody-positive and HCV ribonucleic acid [RNA]-positive). 
  Note: Patients who have documented anti-HCV treatment for a past HCV infection AND are HCV RNA-negative may be enrolled in the study.
16. Have evidence of human immunodeficiency virus (HIV) infection and/or positive HIV antibodies.
17. Have had household contact with a person with active tuberculosis (TB) and did not receive appropriate and documented prophylaxis for TB.
18. Have evidence of active TB or latent TB.
  a Have evidence of active TB, defined in this study as the following: 
    Positive purified protein derivative (PPD) test (≥ 5 mm induration between approximately 2 and 3 days after application, regardless of vaccination history), medical history, clinical features, and abnormal chest X-ray at screening.
     QuantiFERON®-TB Gold test or T-SPOT®.TB test (as available and if compliant with local TB guidelines) may be used instead of the PPD test. Patients are excluded from the study if the test is not negative and there is clinical evidence of active TB.
     Exception: patients with a history of active TB who have documented evidence of appropriate treatment, have no history of re-exposure since their treatment was completed, have no clinical features of active TB, and have a screening chest X-ray with no evidence of active TB may be enrolled if other entry criteria are met. Such patients would not be required to undergo the protocol-specific TB testing for PPD, QuantiFERON®-TB Gold test, or T-SPOT®.TB test but must have a chest X-ray at screening (i.e., chest imaging performed within the past 6 months will not be accepted).
  b Have evidence of untreated/inadequately or inappropriately treated latent TB, defined in this study as the following:
     Positive PPD test, no clinical features consistent with active TB, and a chest X-ray with no evidence of active TB at screening; or
     If the PPD test is positive and the patient has no medical history or chest X-ray findings consistent with active TB, the patient may have a QuantiFERON®-TB Gold test or T-SPOT®.TB test (as available and if compliant with local TB guidelines). If the test results are not negative, the patient will be considered to have latent TB (for purposes of this study); or
     QuantiFERON®-TB Gold test or T- SPOT®.TB test (as available and if compliant with local TB guidelines) may be used instead of the PPD test. If the test results are positive, the patient will be considered to have latent TB. If the test is not negative, the test may be repeated once within approximately 2 weeks of the initial value. If the repeat test results are again not negative, the patient will be considered to have latent TB (for purposes of this study). 
    Exception: Patients who have evidence of latent TB may be enrolled if he or she completes at least 4 weeks of appropriate treatment prior to randomization and agrees to complete the remainder of treatment while in the trial. 
    Exception: Patients with a history of latent TB who have documented evidence of appropriate treatment, have no history of re-exposure since their treatment was completed, have no clinical features of active TB, and have a screening chest X-ray with no evidence of active TB may be enrolled if other entry criteria met. Such patients would not be required to undergo the protocol-specific TB testing for PPD, QuantiFERON®-TB Gold test, or T-SPOT®.TB test but must have a chest X-ray at screening (i.e., chest imaging performed within the past 6 months will not be accepted).
19. Have been exposed to a live vaccine within 12 weeks of randomization or are expected to need/receive a live vaccine during the study (except for herpes zoster vaccination).
20. Are currently enrolled in or have discontinued within 4 weeks of screening from any other clinical trial involving an investigational product or nonapproved use of a drug or device or any other type of medical research judged not to be scientifically or medically compatible with this study.
21. Participants with active renal or central nervous system disease or significant impairment of major organ function (lung, heart, liver, kidney) or any condition that, in the opinion of the Investigator, would jeopardize the participant’s safety following exposure to the study drug.
22. Psychiatric illness or history of medical non-compliance that the study team feels will make the patient unlikely to complete the study.
23. Known allergic reactions to baricitinib or its components.
24. Are largely or wholly incapacitated permitting little or no self-care, such as being bedridden or confined to wheelchair.
25. In the opinion of the investigator, are at an unacceptable risk for participating in the study.
26. Have donated more than a single unit of blood within 4 weeks prior to screening or intend to donate blood during the study.
27. Have a history of intravenous drug abuse, other illicit drug abuse, or chronic alcohol abuse within the 2 years prior to screening or are concurrently using, or expected to use during the study, illicit drugs (including marijuana).
28. Are unable or unwilling to make themselves available for the duration of the study and/or are unwilling to follow study restrictions/procedures.

### Who will take informed consent? {26a}

Consent will be taken by members of the study team who have been delegated the responsibility of taking informed consent by the Principal Investigator. Informed consent will be obtained and documented prior to the participant undergoing study procedures. The informed consent document will comply with Good Clinical Practice (GCP) and local regulatory guidelines.

Informed consent will be obtained in Chinese. The investigator will retain the original of each participant’s signed consent document. The informed consent document used in this study, and any changes made during the study, will be prospectively approved by the Research Ethics Committee/Institutional Review Board (REC/IRB). Participants are free to withdraw at any time from the study without providing a reason.

### Additional consent provisions for collection and use of participant data and biological specimens {26b}

Participants will be noticed and signed the informed consent for collection and storage of clinical data and biological samples. No individual personal data from patients will be contained in collection and use of the biological specimens.

#### Public and patient involvement

There will be no public or patient involvement in the design of the protocol of our study.

## Interventions

### Explanation for the choice of comparators {6b}

Baricitinib has been approved for the treatment for active RA. There are already some reports of baricitinib used in some other autoimmune diseases, such as SLE [20], dermatomyositis [21], and polymyalgia rheumatica/giant cell arteritis [7]. In a double-blind, multi-center, randomized, placebo-controlled, 24-week phase 2 study, baricitinib 4 mg per day significantly improved the signs and symptoms of active SLE, especially arthritis or rash, and showed a safety profile consistent with previous studies in RA [20]. So, we choose the dosage of baricitinib 4 mg per day in this study.

We had already observed that baricitinib treatment for 6 months significantly improved the symptoms and the ESSDAI score of active pSS patients in a pilot study established at PUMCH (see “Discussion”). Based on the basic researches and clinical studies mentioned above, we thought that baricitinib might have therapeutic benefit in patients with active pSS. The conventional treatment of SS includes topical and systemic medications such as HCQ, oral glucocorticoids, and immunosuppressive agents. We choose HCQ and low dose of steroids as the background treatment in our study.

### Intervention description {11a}

Patients with active pSS (ESSDAI ≥ 5) under 12 weeks treatment of HCQ will be involved in the study. Patients will be randomized (2:1) to receive baricitinib 4 mg per day + HCQ 400 mg per day or HCQ 400 mg per day alone. Patients will come to visit at weeks 0, 4, 8, 12, 16, 20, and 24. Patients who have no ESSDAI response to HCQ treatment alone at week 12 will be switched to baricitinib + HCQ group and added on with baricitinib 4 mg per day until the end of the study (week 24). The final evaluation will be at week 24.

### Criteria for discontinuing or modifying allocated interventions {11b}

#### Temporary interruption of investigational product

In some circumstances, patients may need to temporarily interrupt treatment as a result of abnormal laboratory values that may have an unclear relationship to investigational product.

For the abnormal laboratory findings and clinical events (regardless of relatedness) listed in Table 1, specific guidance is provided for temporarily interrupting treatment and when treatment may be restarted. Retest frequency and timing of follow-up laboratory tests to monitor the abnormal finding is at the discretion of the investigator. Investigational product that was temporarily interrupted because of an adverse event or abnormal laboratory value not specifically covered in Table 1 may be restarted at the discretion of the investigator.

**Table 1:** Temporary interruption due to some abnormal laboratory values

Laboratory measure	Action	Monitoring guidance
Absolute neutrophil count (ANC)	Treatment should be interrupted if ANC < 1 × 10^9^ cells/L and may be restarted once ANC return above this value	Before treatment initiation (see exclusion criteria too) and thereafter according to routine patient management
Absolute lymphocyte count (ALC)	Treatment should be interrupted if ALC < 0.5 × 10^9^ cells/L and may be restarted once ALC return above this value	
Hemoglobin (Hgb)	Treatment should be interrupted if Hgb < 8 g/dL and may be restarted once Hgb return above this value	
Hepatic transaminases	Treatment should be temporarily interrupted if drug-induced liver injury is suspected	

### Permanent discontinuation from investigational product

Investigational product must be permanently discontinued if the patient or the patient’s designee requests to discontinue investigational product.

Discontinuation of the investigational product for abnormal liver tests should be considered by the investigator when a patient meets 1 of the following conditions:

- ALT or AST > 8 × upper limit of normal
- ALT or AST > 5 × upper limit of normal for more than 2 weeks after temporary interruption of investigational product
- ALT or AST > 3 × upper limit of normal and total bilirubin level > 2 × upper limit of normal
- ALT or AST > 3 × upper limit of normal with the appearance of fatigue, nausea, vomiting, right upper quadrant pain or tenderness, fever, rash, and/or eosinophilia (> 5%)

Investigational product should be permanently discontinued if any of the following laboratory abnormalities are observed:

- WBC < 1 × 10^9^ cells/L
- ANC < 0.5 × 10^9^ cells/L
- ALC < 0.2 × 10^9^ cells/L
- Hgb < 6.5 g/dL

Temporary interruption rules must be followed where applicable. For laboratory values that meet permanent discontinuation thresholds, investigational product should be discontinued. However, if in the opinion of the investigator the laboratory abnormality is due to intercurrent illness such as cholelithiasis or another identified factor, laboratory tests may be repeated. Only when the laboratory value meets resumption thresholds (Table 1) following the resolution of the intercurrent illness or other identified factors may the investigator restart investigational product.

In addition, patients will be discontinued from investigational product in the following circumstances:

- Pregnancy
- Malignancy
- Development of a VTE (DVT/PE) during the study

#### HBV DNA monitoring

HBV DNA testing will be performed in enrolled patients who tested positive for HBcAb at screening. Patients who are HBcAb-positive and HBV DNA-negative (undetectable) at Visit 1 will require HBV DNA monitoring every 3 months and at the patient’s last visit, regardless of their hepatitis B surface antibody (HBsAb) status. If a result is obtained with a value above limit of quantitation at any time during the study, the patient will be permanently discontinued from investigational product and should be referred to a hepatology specialist immediately.

#### Active TB monitoring

Patients with a history of active or latent TB who have documented evidence of appropriate treatment, have no history of re-exposure since their treatment was completed, have no clinical features of active TB, and have a screening chest X-ray with no evidence of active TB may be enrolled.

These patients will require TB activity monitoring every 3 months and at the patient’s last visit. If the patient had active TB during the study in the opinion of the investigator (according to the clinical features and chest X-ray or CT scan), he/she should be permanently discontinued from investigational product and should be referred to an infectious disease specialist immediately.

### Strategies to improve adherence to interventions {11c}

Adherence to treatment schedules will be assessed by pill counts at study visits by direct questioning of dosing schedules and missed doses, telephone visits, and self-report. Participants shall be followed-up in clinic regularly to receive timely and comprehensive consultation and monitoring in the process of treatment. They are in close contact with the investigators in different centers and monitor progression during study visits. This study will use the Chinese Rheumatism Data Center (CRDC) platform [22] and the application of CRDC to ensure regular and standardized follow-up of every patient.

### Relevant concomitant care permitted or prohibited during the trial {11d}

Except for medication which may be required to treat adverse events, no medication other than study drugs will be allowed from the first dosing until all the end of study evaluations have been conducted.

Participants were allowed to use glucocorticoids but the dosage should be less than or equal to 10 mg prednisone per day. Other immunosuppressants including methotrexate, azathioprine, mycophenolate mofetil, cyclosporine, and tacrolimus were not permitted during the whole study. Plasma exchange and IVIg were prohibited during the study. Drugs that promote salivary secretion, such as anethole trithione and pilocarpine, sodium hyaluronate eye drops, and artificial tears shall not be used within 7 days before randomization and evaluation of exocrine gland function.

### Provisions for post-trial care {30}

The project will provide insurance to all participants for damage caused by the therapies of the study. All participants with post-recruitment illness will be monitored until symptoms resolve, until laboratory changes return to baseline, or until there is a satisfactory explanation for the changes observed. They will receive essential medical care at different centers described above.

### Outcomes {12}

#### Primary outcome measure

The rate of ESSDAI response, or MCII of ESSDAI, which was defined as an improvement of ESSDAI at least three points, at 12 weeks.

#### Secondary outcome measure

1. Rate of MCII of ESSDAI at 24 weeks.
2. Rate of EULAR pSS patient-reported index (ESSPRI) response, or MCII of ESSPRI, which was defined as an improvement of ESSPRI at least one point or 15%, at 12 and 24 weeks.
3. Change of Physician’s Global Assessment (PGA) score from baseline at 12 and 24 weeks.
4. Change of serological activity parameters including CRP, ESR, IgG, and RF levels from baseline at 12 and 12 weeks.
5. Change of salivary glands function including the salivary flow rate (ml/min) and the Schirmer’s test (mm) from baseline at 12 and 24 weeks.
6. Change of focus score on labial salivary gland biopsy [23] from baseline at 24 weeks. The minimum value is 1 and maximum value is 12, and higher scores mean a worse outcome.

### Participant timeline {13}

Table 2 shows the participant timeline. The window period of each visit is 2 weeks.

**Table 2:** The participant timeline

	− 4 weeks (screening)	0 weeks (start medication)	4 weeks	8 weeks	12 weeks (switch checkpoint)	16 weeks	20 weeks	24 weeks (the end of study)
Visit	0	1	2	3	4	5	6	7
informed consent	√							
Medical history record	√							
Vital signs	√	√	√	√	√	√	√	√
Infection screening like hepatitis and TB	√							√
complete blood cell count	√	√	√	√	√	√	√	√
Renal and liver function test	√	√	√	√	√	√	√	√
Urine routine test	√	√	√	√	√	√	√	√
Acute phase reactants (ESR, CRP)	√	√			√			√
Autoantibodies (antinuclear antibody, anti-SSA, anti-SSB, et al.)	√							√
Immunological parameters (Ig, complement, RF)	√				√			√
Exocrine gland function	√				√			√
Labial salivary gland biopsy	√							√
PGA score	√	√			√			√
ESSDAI score	√	√			√			√
ESSPRI score	√	√			√			√
Adverse events	√	√	√	√	√	√	√	√
Concomitant medication	√	√	√	√	√	√	√	√

### Sample size {14}

According to the results of our pilot study [24] and clinical experiments, the expected response rate is 70% (μ) in baricitinib + HCQ group, and 30% (μ0) in HCQ group. We use this formula to calculate the sample size:

$$N=\frac{{\left({z}_{a/2}+{z}_{\beta }\right)}^{2}{\sigma }^{2}}{{\left(\mu -{\mu }_{0}\right)}^{2}}$$

Zα/2 = 1.96 when alpha = 0.05, Zβ = 0.84 when beta = 0.2. After calculation, the sample will be 46:23. And consider the dropout rate as 20%, we will involve approximately 87 patients (58:29) for the study.

### Recruitment {15}

Participants will be recruited at eight different centers distributed throughout seven provinces in China. The leadership center, Peking Union Medical College Hospital (PUMCH), is a referral center for difficult and severe rheumatic and autoimmune diseases in China. We believe that enough patients who meet the inclusion and exclusion criteria can be involved in this study.

## Assignment of interventions: allocation

### Sequence generation {16a}

Eligible participants will be randomized into HCQ group or baricitinib + HCQ group with a computer-generated randomization schedule operated at the application of CRDC.

### Concealment mechanism {16b}

Allocation is not concealed and will be revealed to both the patient and the researcher upon randomization.

### Implementation {16c}

Confirmation of eligibility and enrolment of participants into the trial will be assigned to a medical practitioner. After signing the informed consent forms, the researchers will use the computer-generated randomization schedule to allocate the patient to one of the study arms. The study group will be revealed at the same time to both the patient and researcher.

## Assignment of interventions: blinding

### Who will be blinded {17a}

Because this is an open-label study, all the participants and researchers will not be blinded.

### Procedure for unblinding if needed {17b}

The trial design is open label; therefore, there is no unblinding procedure.

## Data collection and management

### Plans for assessment and collection of outcomes {18a}

Data will be collected and saved in the hospital information system (HIS) in each single center. The study will use the CRDC platform and the application of CRDC to follow-up patients. The mobile device application for the CRDC ensures convenient and standardized clinical data collection for this study.

### Plans to promote participant retention and complete follow-up {18b}

The participants will receive extensive information about the study during the recruitment. The importance of completion of the follow-up will be stressed. However, the participants may voluntarily withdraw from the study for any reason at any time. We will use the application of CRDC to remind participants to follow-up regularly. They can also contact the study team if they have any questions of the study at any times.

For participants who fail to appear for study visits without stating an intention to withdraw, the study team will try to contact the participant through mobile device application, telephone calls, or short messages. If a participant withdrawal occurs for any reason, the study team must record the primary reason for a subject’s withdrawal from the study. If a participant withdraws from the study and disclosure of future information, no further evaluations will be performed, and no additional data will be collected. The investigator may retain and continue to use any data collected before such withdrawal of consent.

### Data management {19}

Data will be collected with electronic case report forms (eCRFs) and stored securely in the database of CRDC. Informed consent and signed paper forms will be stored within every center in a locked room with access granted only to authorized study staff. Source data will remain available in electronic patient record. All research data, including patient medical record, informed consent, and other related materials, will be archived for at least 5 years after the study.

### Confidentiality {27}

Research data including eCRFs will be stored securely into the database of CRDC. Access to the database will only be available to the research team during the study and will be documented and safeguarded by the principal investigator according to research guidelines after completion of the study. Other trial documents including the informed consent will be kept in locked cabinets. No participant identifying information will be disclosed in any publication or at any conference activities arising from the study.

### Plans for collection, laboratory evaluation, and storage of biological specimens for genetic or molecular analysis in this trial/future use {33}

As mentioned above, JAK/STAT pathway may take part in the process of pSS by affecting many cytokine signals. Because there are few studies about baricitinib in pSS, the exploration for the mechanism of JAK/STAT inhibitor in the treatment of pSS is important for further research. Therefore, we plan to collect the residue of blood and tissue specimens which were used for laboratory evaluation at every visit for further basic research such as genetic or molecular analysis in the future. Participants will be noticed and signed the informed consent.

## Statistical methods

### Statistical methods for primary and secondary outcomes {20a}

Data will be analyzed using IBM SPSS 23.0 (Chicago, IL, USA). Differences will be considered statistically significant if *p* < 0.05. Continuous variables were analyzed using paired *t*-tests for data comparison between the two treatment groups. Categorical variables were analyzed using the chi-square test or Fisher’s exact test, where appropriate. Two-tailed tests were used for all analyses, and *P* < 0.05 was considered statistically significant. The 95% confidence interval of the fixed effect size will be used to assess whether treatment difference reaches the minimally clinical important difference. The changes in outcome measures among all participants at 24 weeks will be used for intention-to-treat analyses.

### Methods for additional analyses {20b}

There are no subgroup analyses and adjusted analyses planned.

### Interim analyses {21b}

Patients who have no ESSDAI response to HCQ treatment alone at week 12 will be switched to baricitinib + HCQ group and added on with baricitinib 4 mg per day until the end of the study (week 24). There are no interim safety analyses planned for this trial.

### Methods in analysis to handle protocol non-adherence and any statistical methods to handle missing data {20c}

No imputation of missing data will be considered for the efficacy and analysis in this trial.

### Plans to give access to the full protocol, participant-level data, and statistical code {31c}

The full protocol, anonymous participant-level dataset, and statistical code of this study can be made available by the corresponding author upon reasonable request and according with the research collaboration agreement.

## Oversight and monitoring

### Composition of the coordinating center and trial steering committee {5d}

This is a multiple center study performed in eight different centers. The leading site is Peking Union Medical College hospital (PUMCH). Day to day support for the trial is provided by:

- Principle investigator: takes supervision of the trial and medical responsibility of the patients.
- Data manager: organizes data capture, safeguards quality and data.
- Study physician: identifies potential recruits, takes informed consent, ensures follow-up and safety monitor according to protocol.

There is no trial steering committee or stakeholder and public involvement group.

### Composition of the data monitoring committee, its role, and reporting structure {21a}

Since the two drugs used in this study, baricitinib and hydroxychloroquine, were all approved in China and many other countries, there is no Data Safety Monitoring Board (DSMB) committee for the safety monitoring in this study. However, this study was approved by the Institutional Review Board (IRB) of Peking Union Medical College Hospital (PUMCH) and other sites. In case of SAEs, the researchers will report to IRB and State Food and Drug Administration of China within 24 h. Moreover, since this is not a blinded study, there is no DSMB required to protect blinding of the researchers and physicians.

### Adverse event reporting and harms {22}

An adverse event (AE) is any untoward medical occurrence (including an abnormal laboratory finding), in a patient or clinical trial subject administered a medicinal product temporally associated with the use of a study agent(s), whether or not related to the study agent(s), occurring as soon as the patient has signed the informed consent form and at any time during the study. All adverse events reported by the subject or observed by the investigators will be recorded in the CRF. AEs also include an undesirable medical condition occurring, even if no study treatment has been administered.

For all AEs, the investigator will assess the causal relationship between the study drug and the AE using his/her clinical expertise and judgment according to the following algorithm that best fits the circumstances of the AE:

The investigator will interpret and document whether or not an AE has a reasonable possibility of being related to study treatment, study device, or a study procedure, taking into account the disease, concomitant treatment or pathologies. The investigator will decide whether the patient will drop out of the study due to the AEs.

A “reasonable possibility” means that there is a cause and effect relationship between the investigational product, study device, and study procedure and the AE. The investigator answers yes/no when making this assessment.

A serious AE (SAE) is any AE from this study that results in one of the following outcomes: death; initial or prolonged inpatient hospitalization; a life-threatening experience (that is, immediate risk of dying); persistent or significant disability/incapacity; congenital anomaly/birth defect; important medical events that may not be immediately life-threatening or result in death or hospitalization but may jeopardize the patient or may require intervention to prevent one of the other outcomes listed in the definition above.

Record the incidence of AEs and SAEs, especially which may cause discontinuation of the study.

Attention should be paid to monitoring the risk of serious infections such as tuberculosis, hepatitis B and other infections after medication. The risk of thromboembolic events should be monitored. Other adverse events included abnormal blood routine test, abnormal liver and kidney function test, allergic reaction, and gastrointestinal reaction.

### Frequency and plans for auditing trial conduct {23}

Periodic monitoring will be conducted independent of the investigators by monitors from the IRB and department of rheumatology in PUMCH. The independent monitor makes two on-site visits per year and checks the presence and completeness of the investigation file. The monitor checks the following data for 25% randomly picked patients: informed consents, inclusion and exclusion criteria, source data, and missing and reporting for AEs and SAEs.

### Plans for communicating important protocol amendments to relevant parties (e.g., trial participants, ethical committees) {25}

Protocol deviations will be communicated to the appropriate authorities within seven days. Amendments to the protocol will be implemented following local ethics and regulatory approvals and updated in ClinicaTrials.gov.

### Dissemination plans {31a}

Results of this research will be disclosed completely in international peer-reviewed journals. Both positive and negative results will be reported. Participants wanting to see the results of the trial can request a copy of the article from the investigators once it has been published. Full anonymity of participant’s details will be maintained throughout.

## Discussion

Baricitinib, a JAK1 and JAK2 inhibitor, was approved for the treatment of RA and widely used in many other autoimmune diseases. Baricitinib has been reported to be effective for treating cutaneous involvement in SLE, including refractory skin papulosquamous rash [25] and diffuse non-scarring alopecia [26]. The mechanism might be associated with inhibition of the IFN signaling pathway [27]. The phase 2 study of baricitinib for SLE has also shown the potential therapeutic efficacy [20]. This study also demonstrated that baricitinib treatment reduced mRNA expression of multiple IFN responsive genes and consistently suppressed two key cytokines implicated in SLE pathogenesis, IL-12p40 and IL-6 [28]. JAK inhibitors might be helpful for the treatment of pSS throughout the IFN pathway, as well as the other autoimmune diseases. But both the basic and clinical evidences are limited.

This is the first randomized controlled study to evaluate the clinical efficacy and safety of baricitinib in pSS. We have already finished the pilot study of baricitinib in active pSS patients. In the pilot study, we involved 11 pSS patients with ESSDAI ≥ 5 and treated them with baricitinib 2 mg per day on the basis of the original treatment. We found that baricitinib significantly improved the ESSDAI score and might be helpful for the management of various manifestations of pSS, such as constitutional symptoms, arthritis, skin rash, hematological involvement, and even interstitial lung disease [24]. Since it is a pilot study with no controlled group, and we did not test the dosage of 4 mg baricitinib per day in pSS, which was involved in the clinical trial of SLE [20], and proven to be effective and safe for the management of RA [29], we designed this prospective, randomized controlled study to better evaluate the potential therapeutic efficacy of baricitinib in pSS. We hope that the result of this study can provide more reliable evidence of the efficacy and safety of baricitinib in pSS. It might also lead us to explore novel therapeutic option such as other JAK inhibitors in pSS.

## Trial status

The initiation meeting of this clinical trial convened at February 12th, 2022. Screening of participants into the study with protocol Version 3.1, January 30th, 2022, began on February 12th, 2022.

## Data Availability

The datasets used and/or analyzed during the current study will be made available from the corresponding author upon reasonable request.

## Abbreviations

pSS: Primary Sjogren’s syndrome
JAK/STAT: Janus kinase/signal transduction and activator of transcription
HCQ: Hydroxychloroquine
EULAR: European League Against Rheumatism
ESSDAI: EULAR pSS disease activity index
MCII: Minimal clinically important improvement
ESSPRI: EULAR pSS patient-reported index
PGA: Physician’s Global Assessment
Th: T helper
IL: Interleukin
TGF: Transforming growth factor
GWAS: Genome-wide association study
SLE: Systemic lupus erythematosus
RA: Rheumatoid arthritis
BAFF: B-cell activating factor
IFN: Interferon
IRB: Institutional review board
ERB: Ethics review board
CRP: C-reactive protein
ESR: Erythrocyte sedimentation rate
IgG: Immunoglobulin G
RF: Rheumatoid factor
IVIg: Intravenous immunoglobulin
ALT: Alanine aminotransferase
AST: Aspartate aminotransferase
eGFR: Estimated glomerular filtration rate
WBC: White blood cell
ANC: Absolute neutrophil count
Hgb: Hemoglobin
ALC: Absolute lymphocyte count
TSH: Thyroid-stimulating hormone
VTE: Venous thromboembolism
DVT: Deep vein thrombosis
PE: Pulmonary embolism
HBV: Hepatitis B virus
HBsAg: Hepatitis B surface antigen
HBcAb: Hepatitis B core antibody
HBV DNA: Hepatitis B virus deoxyribonucleic acid
HCV: Hepatitis C virus
HCV RNA: Hepatitis C virus ribonucleic acid
HIV: Human immunodeficiency virus
TB: Tuberculosis
PPD: Purified protein derivative
GCP: Good clinical practice
PUMCH: Peking Union Medical College Hospital
HBsAb: Hepatitis B surface antibody
CRDC: Chinese Rheumatism Data Center
HIS: Hospital information system
eCRF: Electronic case report form
DSMB: Data Safety Monitoring Board
AE: Adverse event
SAE: Serious AE
