# Correction: A multi-center, open-label, randomized study to explore efficacy and safety of baricitinib in active primary Sjogren’s syndrome patients

**DOI:** 10.1186/s13063-023-07546-z

**Published:** 2023-07-31

**Authors:** Wei Bai, Fan Yang, Huji Xu, Wei Wei, Hongbin Li, Liyun Zhang, Yi Zhao, Xiaofei Shi, Yan Zhang, Xiaofeng Zeng, Xiaomei Leng

**Affiliations:** 1 Department of Rheumatology and Clinical Immunology, Peking Union Medical College Hospital, Chinese Academy of Medical Sciences, Peking Union Medical College; National Clinical Research Center for Dermatologic and Immunologic Diseases (NCRC-DID), Ministry of Science & Technology; State Key Laboratory of Complex Severe and Rare Diseases, Peking Union Medical College Hospital; Key Laboratory of Rheumatology and Clinical Immunology, Ministry of Education, Beijing, 100730 China; 2 Department of Rheumatology and Immunology, Changzheng Hospital, Naval Medical University, Shanghai, 200003 China; 3 Department of Rheumatology and Immunology, Tianjin Medical University General Hospital, Tianjin, China; 4 Department of Rheumatology, The Afliated Hospital of Inner Mongolia Medical University, Hohhot, Inner Mongolia China; 5 Department of Rheumatology, Third Hospital of Shanxi Medical University, Bethune Hospital Shanxi Academy of Medical Sciences, Taiyuan, Shanxi China; 6 Department of Rheumatology and Allergy, Xuanwu Hospital, Capital Medical University, Beijing, China; 7 Department of Rheumatology and Immunology, The First Afliated Hospital and College of Clinical Medicine, Henan University of Science and Technology, Luoyang, Henan China; 8 Department of Rheumatology and Immunology, Tangdu Hospital, Fourth Military Medical University (Air Force Medical University), Xi’an, Shaanxi China

**Correction: BMC Trials 24, 112 (2023)**

**https://doi.org/10.1186/s13063-023-07087-5**

Following publication of the original article [1], we have been notified that affiliations 1 to 4 are actually different titles within the same department and should be gathered under just one affiliation.

Additionally, the following sentence was added in the Funding section: This study was supported by the National High Level Hospital Clinical Research Funding (2022-PUMCH-A-227).

The original article has been corrected.
